# Going beyond efficacy: supportive care and addressing the toxicity of systemic therapy

**DOI:** 10.3747/co.2008.175

**Published:** 2008-01

**Authors:** S. Verma

Significant advances in oncology have been made in recent decades. In general, the mortality rates associated with most cancers are now decreasing, and a significant number of new treatments are now available for cancer patients. As oncologists use more complex treatments to cure patients or to prolong their survival, recognition and management of treatment-related toxicity takes on critical importance.

In this issue of *Current Oncology,* subtitled “Going Beyond Efficacy,” leaders from across the country focus on some serious issues that are being faced by cancer patients. We are delighted to have involvement from many different specialties, highlighting the multidisciplinary nature of cancer management.

- A common short-term toxicity of cancer treatment is nausea and vomiting. Dr. David Warr addresses this issue and discusses recent advances in the management of this troublesome symptom.
- As the complexity of treatments increases, and as combination chemotherapy becomes more common, treatment-related anemia and neutropenia need to be addressed. Dr. Barb Melosky presents up-to-date information on anemia in cancer patients and specifically addresses recent guidelines for, and safety concerns with, erythropoiesis-stimulating agents. (An upcoming article in the journal will also address neutropenia.)
- As therapies become more complex and as cure rates improve in early-stage settings, the long-term sequelae of adjuvant treatment of cancer must be considered. A common concern in the patient population with breast cancer is the risk of cardiac toxicity associated with cancer treatment. Dr. Kathryn Towns, Dr. Philippe Bedard, and I present data on the risk of cardiac toxicity associated with adjuvant therapy in breast cancer.
- Another significant concern related to cancer treatment is the development of bone loss. Dr. Aliya Khan provides an endocrinologist’s perspective of bone loss in relation to cancer treatment—its implication and its management.
- In the metastatic setting, one of the main goals of treatment is to provide optimal palliation. Dr. Jeff Myers, a palliative care physician, discusses the basics of cancer pain management and also presents information on new medical and interventional therapies.
- In advanced disease, bone metastases commonly occur. Dr. Bianca Petrut and colleagues provide a primer on the management of bone metastases in breast cancer.
- Thrombosis presents a serious risk of complication in cancer patients. Dr. Agnes Lee discusses the most recent evidence on the prevention and treatment of venous thromboembolism in cancer patients.

These authors and I all hope that readers will find this supplementary issue of *Current Oncology* to be clinically meaningful as they focus on “going beyond efficacy” to address some of the serious issues faced by cancer patients.

